# WH01-3A: a DIVA-compliant, ApxIA, ApxIIA, and ApxIIIA expressing *Actinobacillus pleuropneumoniae* live attenuated vaccine strain that protects mice and pigs against homologous and heterologous serovars

**DOI:** 10.1186/s13567-026-01819-6

**Published:** 2026-07-30

**Authors:** Lu Peng, Qiuhong Zhang, Qingyi Zhou, Weiyao Han, Delan Yang, Zhen Luo, Zhichang Liu, Rui Zhou, Yunfeng Song, Paul R. Langford, Lu Li

**Affiliations:** 1 National Key Laboratory of Agricultural Microbiology, College of Veterinary Medicine, Huazhong Agricultural University, Shizishan Street 1, Wuhan, 430070 Hubei China; 2 Key Laboratory of Preventive Veterinary Medicine in Hubei Province, The Cooperative Innovation Center for Sustainable Pig Production, Wuhan, 430070 Hubei China; 3 Section of Paediatric Infectious Disease, Imperial College London, South Kensington Campus, London, SW7 2AZ UK; 4 International Research Center for Animal Disease, Ministry of Science and Technology of the People’s Republic of China, Wuhan, 430070 Hubei China; 5 Shandong Binzhou Institute of Animal Husbandry and Veterinary Science, Binzhou, 256600 Shandong China

**Keywords:** *Actinobacillus pleuropneumoniae*, live attenuated vaccine, Apx toxins, immune protection

## Abstract

*Actinobacillus pleuropneumoniae* is the causative agent of porcine pleuropneumonia, a major economic burden to the worldwide swine industry. Many available vaccines protect against one or a few of the 19 serovars known. Live attenuated vaccines can potentially protect against many different serovars. In this study, the highly virulent parental *A. pleuropneumoniae* serovar 1 strain WH01 was used to construct WH01-3A, expressing nontoxic (nonacylated) versions of the three major toxin antigens ApxIA, ApxIIA, and ApxIIIA. In addition, the vivo-induced toxin ApxIVA was deleted, enabling use in a differentiation of vaccinated from infected animals (DIVA) strategy. Compared with WH01, the virulence of WH01-3A in mice was significantly reduced, and, when used as a live attenuated vaccine, WH01-3A protected 87.5%, 75%, and 87.5% animals against *A. pleuropneumoniae* challenge with serovars 1, 5, and 15, respectively. Pigs immunized with up to 5 × 10^8^ CFU WH01-3A had normal body temperatures and behaviors, and normal lung and tonsil pathology. WH01-3A immunization of pigs at 1 × 10^8^ CFU resulted in 66.7% and 100% protection after challenge with *A. pleuropneumoniae* serovars 1 and 15, respectively. WH01-3A immunization induced antibodies against ApxI, ApxII, ApxIII, and whole-cell proteins of serovars 1 and 15. Compared to the unvaccinated group, the average lung lesion scores were reduced by 42.6% and 67.8% after challenge with serovars 1 and 15, respectively. We conclude that WH01-3A is a safe and effective live attenuated vaccine, providing effective immunological protection against *A. pleuropneumoniae* serovars 1 and 15.

**Supplementary Information:**

The online version contains supplementary material available at 10.1186/s13567-026-01819-6.

## Introduction

Porcine pleuropneumonia caused by *Actinobacillus pleuropneumoniae* is a worldwide, economically important swine respiratory disease. The pathological features include hemorrhagic, fibrinous, and necrotic lung lesions. Acute infections often result in high mortality rates [1]. On the basis of capsular polysaccharide, 19 serovars of *A. pleuropneumoniae* have been identified [2]. Serovar prevalence varies between countries and regions, and changes over time [3–7]. For example, serovar 15, originally described in Australia [8]. Our laboratory has collected 90 recent *A. pleuropneumoniae* isolates from 20 provinces in China, and serovar 15 was the most frequently isolated, followed by 1 and 7 [9]. Serovar 15 isolates have also been described from Japan [10]. Although less commonly reported in North America, where serovars 7 and 8 predominate, serovar 15 was reported to affect multiple companies in Iowa, USA [11]. Strains were unexpectedly highly pathogenic, geographic and temporally persistent, and had a propensity for lateral spread [11]. Pigs persistently infected with *A. pleuropneumoniae* are a major threat to farms. Although these pigs show no obvious symptoms, they can carry the pathogen for extended periods and continuously shed it through direct contact or aerosols [12], making it difficult to eradicate the pathogen from the herd. Under stress conditions such as improper management or co-infection, acute outbreaks can be triggered, resulting in severe economic losses [11, 13].

Antibiotic treatment and vaccination are the main measures for treatment and control of *A. pleuropneumoniae*, respectively. However, the long-term use of antibiotics can lead to the emergence of multidrug-resistant strains or increased resistance to commonly used antibiotics such as florfenicol, which is detrimental to the sustainable development of the pig industry [4, 14]. Therefore, vaccination is important for controlling *A. pleuropneumoniae*. Inactivated vaccines generally target homologous serovars and provide relatively limited protection against heterologous serovars [15]. Commercially available inactivated vaccines include products targeting serovars 2 and 6 or serovars 1, 5, and 7 of *A. pleuropneumoniae* [16]. In China, a trivalent inactivated vaccine targeting *A. pleuropneumoniae* serovars 1, 2, and 7 is available. The development of subunit vaccines typically relies on the identification of *A. pleuropneumoniae* virulence factors, including Apx toxins, capsular polysaccharides, lipopolysaccharides, and fimbriae [17]. Among these, Apx toxins are the most important candidate antigens owing to their overlapping distribution among different serovars and their crucial role in inducing protective immunity against porcine pleuropneumonia [18, 19]. Apx toxins combined with other antigens or inactivated bacterial cells can provide effective protection against *A. pleuropneumoniae* challenge [20–22]. The commercial subunit vaccine Porcilis^®^ APP (ApxI + ApxII + ApxIII + OMP) has been widely applied and provides broad-spectrum cross-protection against multiple serovars [23].

Because live attenuated vaccines retain the essentially intact structure of the bacterial strain, they are expected to provide broader protection [15, 24]. ApxI-III toxins are key virulence factors of *A. pleuropneumoniae*, exhibiting distinct hemolytic and cytotoxic activities [18]. ApxI and ApxIII are encoded by complete *apxICABD* and *apxIIICABD* operons, respectively, in which the protein encoded by the *C* gene activates the activity of the structural protein encoded by the *A* gene, and the proteins encoded by the *BD* genes are responsible for the toxin secretion. ApxII is encoded by the *apxIICA* operon, and its secretion relies on the *BD* transport system of ApxI [18, 25]. Additionally, ApxIV, which is expressed in vivo as a natural infection marker, is encoded by the genes *orf1* and *apxIVA* (structural gene) and is crucial for the virulence of *A. pleuropneumoniae* [26, 27]. In a serovar 10 strain of *A. pleuropneumoniae,* deletion of a C-terminal fragment of the *apxIC* gene and insertion of a chloramphenicol resistance cassette resulted in a mutant that expresses a protein of c. 48 kDa comprising the N-terminus of the ApxI toxin. When used as a live attenuated vaccine, the strain conferred cross-protection against serovars 1 and 2 in pigs [28]. Introduction of a shuttle vector carrying the complete *apxIA* gene into an *apxIIC* deletion mutant generated a serovar 7 attenuated strain that co-expresses *apxIA*. This strain induced antibodies against both ApxIA and ApxIIA in pig serum and conferred protection against heterologous challenge with the highly virulent serovar 1 strain [29]. The quintuple deletion mutant SLW07 (∆*apxIC*∆*apxIIC*∆*orf1*∆*cpxAR*∆*arcA*) showed reduced adhesion and invasion of host cells, stimulated Th2-type responses in mice, and induced splenic lymphocytes to produce IL-6 and TNF-α. It provided complete protection in mice and exhibited lower virulence [30].

To date, no natural or constructed strain for vaccine development expressing all three Apx toxins (ApxIA + ApxIIA + ApxIIIA) has been reported. In this study, the highly virulent and prevalent domestic *A. pleuropneumoniae* serovar 1 strain WH01 was selected as the parent. Key virulence genes *apxIVA*, *apxIC*, and *apxIIC* were deleted to attenuate virulence, while the *apxIIIA* gene was inserted into the genome, driven by the promoter of the operon encoding nitrate reductase [31], enabling expression of ApxIA, ApxIIA, and ApxIIIA in a single mutant (WH01-3A). The safety and ability of WH01-3A to protect against challenge with virulent clinical isolates of serovars 1 and 15 were determined in both mice and pigs, providing evidence for its application as a novel live attenuated vaccine.

## Materials and methods

### Bacterial strains and growth conditions

The strains, plasmids, and primers used in this study are listed in Additional file 1. *A. pleuropneumoniae* serovar 1 WH01, serovar 5 202,203,179, and serovar 15 XB2T-56 are clinical isolates preserved in our laboratory collection. *A. pleuropneumoniae* serovar 3 reference strain JL03 [32] (GenBank accession: NC_010278.1), serovar 7 reference strain AP76 (GenBank accession: NC_010939.1), serovar 10 reference strain D13039 (GenBank accession: NZ_CP031864.1), kindly provided by Dr P. Blackall (Queensland Department of Primary Industries, Animal Research Institute, Australia). *A. pleuropneumoniae* were cultured in Tryptic Soy Broth (TSB, Becton Dickinson and Company, NJ, USA) supplemented with 10 μg/mL NAD (Biosharp, Anhui, China) or on Tryptic Soy Agar (TSA) plates containing the same supplement. *Escherichia coli* strain β2155 was cultured in Luria–Bertani (LB) broth (Oxoid, Hampshire, United Kingdom) or on LB agar plates, supplemented with 20 μg/mL chloramphenicol (BioFroxx, Einhausen, Germany) and 50 μg/mL diaminopimelic acid (DAP) (Biosharp, Anhui, China). *A. pleuropneumoniae* mutants were selected on TSA plates containing 10% sucrose (Biosharp, Anhui, China) and 10 μg/mL NAD.

### Construction of genetically engineered mutants

Using WH01 genomic DNA as the template, primers *apxIVA*-1/2 and *apxIVA*-3/4 were used to PCR-amplify upstream and downstream fragments of the *apxIVA* gene, respectively. The amplification products were ligated into the *SalI* and *NotI* sites of the suicide vector pEMOC2 [33] using the ClonExpress MultiS One Step Cloning Kit (Vazyme, Nanjing, China). The recombinant plasmid pEMOC2-Δ*apxIVA* was transformed into *E. coli* β2155 and then transferred into WH01 by coincubation conjugation [34]. WH01 Δ*apxIVA* mutants were selected on plates containing 10% sucrose and confirmed by PCR using the *apxIVA*-UF/DR and Δ*apxIVA*-F/R primers. A confirmed WH01 Δ*apxIVA* mutant was used as the backbone for subsequent further deletions by the same method as detailed below. Primers *apxIC*-1/2 and *apxIC*-3/4, and *apxIIC*-1/2 and *apxIIC*-3/4, were used to amplify the upstream and downstream fragments of the *apxIC* and *apxIIC* genes, respectively, using WH01 genomic DNA as template. Recombinant plasmids pEMOC2-Δ*apxIC* and pEMOC2-Δ*apxIIC* were constructed and separately introduced into *E. coli* β2155. Through coincubation conjugation, a Δ*apxIVA*Δ*apxIC* mutant was generated in the WH01 Δ*apxIVA* background, which was verified by PCR using *apxIC*-UF/DR and Δ*apxIC*-F/R primers. The *apxIIC* gene was then deleted from the WH01 Δ*apxIVA*Δ*apxIC* genome to produce the WH01 Δ*apxIVA*Δ*apxIC*Δ*apxIIC* mutant, which was confirmed by PCR using the *apxIIC*-UF/DR and Δ*apxIIC*-F/R primers. Finally, using WH01 Δ*apxIVA*Δ*apxIC*Δ*apxIIC* genomic DNA as the template, the upstream and downstream fragments flanking the *apxIVA* deletion site were amplified with primers Δ*apxIVA*-1/2 and Δ*apxIVA*-3/4, respectively, while the promoter of the *nap* gene was amplified with primers P*nap*-1/2. The *apxIIIA* gene fragment was amplified from the *A. pleuropneumoniae* serovar 3 strain JL03 using primers *apxIIIA*-1/2. The Δ*apxIVA* upstream fragment, P*nap*, *apxIIIA* gene fragment, and Δ*apxIVA* downstream fragment were simultaneously ligated into the pEMOC2 plasmid digested with *SalI*/*NotI*. The recombinant plasmid pEMOC2-P*nap*/*apxIIIA* was introduced into the WH01 Δ*apxIVA*Δ*apxIC*Δ*apxIIC* strain by coincubation conjugation. After selection, the mutant strain WH01 Δ*apxIVA*Δ*apxIC*Δ*apxIIC*/*apxIIIA*^+^ (designated as WH01-3A) was obtained, confirmed by PCR using Δ*apxIVA*-F/R and P*nap*-F/*apxIIIA*-R primers. WH01-3A genomic DNA was extracted and used as template for PCR reactions using the primers listed in Additional file 1, which confirmed deletion of the *apxIVA*, *apxIC*, and *apxIIC* genes, and insertion of the P*nap*/*apxIIIA* gene.

### RT-PCR and genetic stability determination of WH01-3A

Total RNA was extracted from *A. pleuropneumoniae* JL03 (a serovar 3 clinical isolate processing the *apxIIIA* gene), Δ*apxIV*Δ*apxIC*Δ*apxIIC*, and WH01-3A using the TRIzol lysis reagent, according to the protocol recommended by the manufacturer (Total RNA Extraction Kit; Tianmo Biotechnology Co., Ltd., Beijing, China). The PrimeScript RT reagent kit and gDNA Eraser (Takara, Beijing, China) were used to remove residual gDNA and to synthesize cDNA. The resulting cDNA was used as a template for PCR identification with the primer pair *apxIIIA*-F/*apxIIIA*-R listed in Additional file 1. To assess genetic stability, an overnight culture of the WH01-3A mutant was transferred into fresh TSB medium at a 1:100 dilution and incubated at 37 °C with shaking at 180 rpm for 12 h as the first generation culture. This process was repeated for 20 generations. Genomic DNA was extracted from each generation, and used as template for PCRs using the primers listed in Additional file 1, enabling the genetic stability of deleted and inserted genes in WH01-3A to be determined.

### Western blot analysis

Natural toxin proteins were extracted from the culture supernatants of the serovar 1 strain WH01, serovar 7 reference strain AP76, serovar 10 reference strain D13039 and the WH01-3A mutant, following the method described in reference [35]. To obtain control antigens, the recombinant plasmid pQE80L-ApxIA, and pET-32a-ApxIIA, pQE80L-ApxIIIA, constructed and preserved in our laboratory, was transformed into *E. coli* BL21(DE3) competent cells. Recombinant protein expression was induced at 18 °C for 12 h with 0.5 mM isopropyl-β-D-thiogalactoside (IPTG) to promote inclusion body formation. Following centrifugation, the bacterial cells were disrupted, and the resulting inclusion body pellets were washed three times with wash buffer (50 mM Tris–HCl (pH 8.0), 300 mM NaCl, 10 mM ethylenediaminetetraacetic acid (EDTA), 0.5% Triton X-100). The pellets were then dissolved in denaturation buffer (6 M guanidine hydrochloride, 50 mM Tris–HCl (pH 8.0), 100 mM NaCl, 10 mM dithiothreitol (DTT)). The supernatant containing the solubilized protein was collected and subjected to stepwise dialysis for protein refolding. Protein concentration was determined using the bicinchoninic acid assay. Subsequently, purified recombinant ApxIA, ApxIIA, and ApxIIIA proteins was administered intramuscularly to 6-week-old female BALB/c mice at a dose of 50 μg per mouse (*n* = 5). Following three consecutive immunizations at 14 day intervals, serum was collected to obtain ApxIA-, ApxIIA-, and ApxIIIA- specific antiserum, hereafter referred to as anti-ApxIA, anti-ApxIIA, and anti-ApxIIIA mouse serum, respectively. The natural toxin extracts from the WH01-3A mutant, the corresponding natural toxin extracts from each serovar strain (WH01, AP76, D13039), and the respective recombinant ApxIA, ApxIIA, and ApxIIIA proteins (serving as positive controls) were separated by SDS-PAGE, transferred onto a polyvinylidene difluoride (PVDF) membrane, which was blocked with 5% skimmed milk at 37 °C for 2 h. After washing, the PVDF membrane was incubated overnight at 4 °C with the above prepared mouse antiserum (working dilution of 1:5000). Subsequently, the washed membrane was incubated with horseradish peroxidase (HRP)-conjugated goat anti-mouse IgG antibody (Proteintech, Wuhan, China; diluted 1:10,000) at 37 °C for 1 h. Visualization was performed using an enhanced chemiluminescence (ECL) detection system (Tanon, Shanghai, China).

### In vitro biological characteristics of WH01-3A

Overnight cultures of WH01 and WH01-3A were transferred into fresh TSB medium at a 1:100 dilution and incubated at 37 °C with shaking at 180 rpm. The optical density at 600 nm (OD_600_) was measured every hour to monitor bacterial growth. Both WH01 and WH01-3A were also plated onto TSA plates supplemented with 5% defibrinated sheep erythrocytes and incubated at 37 °C for 16 h. Clear zones around colonies indicated hemolysis. For biofilm formation assays, overnight cultures of WH01 and WH01-3A were transferred into 2 mL fresh Brain–Heart Infusion (BHI) broth (OXOID, UK) supplemented with 10 μg/mL NAD at a 1:75 dilution, and incubated in 12-well culture plates at 37 °C for 5 h. Biofilm formation in each well was quantitatively assessed using 0.1% crystal violet ammonium oxalate Solution (Beyotime, Shanghai, China), as described previously [36].

### Animal experiments

In this study, 4-week-old KM mice (outbred Kunming mice) and six-week-old BALB/c mice which were specific pathogen-free (SPF) females were purchased from the Laboratory Animal Center of Huazhong Agricultural University, Wuhan, China. The mice were of comparable age and body weight and were randomly assigned to different groups after an acclimatization period. In the pig experiments, healthy piglets aged 40 and 55 days were used, with efforts made to balance sex, body weight, and litter of origin as much as possible among groups. All pigs were purchased from a farm in Wuhan that had been under long-term surveillance and confirmed to be negative for *A. pleuropneumoniae* and major pig pathogens. No *A. pleuropneumoniae* vaccines had been used on this farm for an extended period. After arrival at the experimental facility, serum samples were collected 2 days before the start of the experiment. According to the manufacturer’s instructions, a commercial ApxIVA ELISA kit (JNT, Beijing, China) was used again to detect APP-ApxIVA antibodies, confirming that all experimental pigs were antibody-negative prior to the study. The assay was performed as an indirect ELISA, and the S/P value was calculated as follows: S/P = (S − N)/(P − N), with S/P > 0.4 considered positive. Only animals confirmed to be negative were included in the study*.*

Animals were randomly allocated to groups after their health status had been confirmed. No animals were excluded during the study for reasons unrelated to the experimental procedures. The sample sizes (mice: *n* = 6 ~ 8/group; pigs: *n* = 3 ~ 6/group) were determined in accordance with the 3R principles. In addition, considering the cost and welfare implications of using pigs as large experimental animals, a reasonable number of animals suitable for statistical analysis was selected, and *n* = 3 was used for the blank control group to exclude potential environmental confounding factors.

### Virulence determination in mice

Sixty-six 4-week-old female KM mice were randomly divided into 11 groups (*n* = 6). Five groups were challenged intraperitoneally with 200 μL of WH01 bacterial cultures at doses ranging from 1 × 10^6^ to 9.5 × 10^6^ CFU (Table 1). Another five groups were similarly infected with the WH01-3A mutant, at doses ranging from 2.5 × 10^8^ to 1 × 10^9^ CFU (Table 1). The control group was injected intraperitoneally with 200 μL of PBS. Mice were observed continuously for 72 h post-challenge. The 50% lethal dose (LD_50_) values for the WH01 and WH01-3A strains were calculated using the Reed–Muench method [37]. However, given the small sample size per group (*n* = 6), the precision of these estimates may be limited. Accordingly, the LD_50_ values need to be interpreted as measurements of relative virulence rather than absolute values.

**Table 1:** **Virulence of** ***A. pleuropneumoniae* WH01 and WH01-3A in mice**

Strains	Challenge dose (CFU)	Number of deaths	Mortality rate	Value of LD_50_ (CFU)
WH01	1 × 10^6^	0	0	4.9 × 10^6^
	2.5 × 10^6^	1	16.7	
	4.5 × 10^6^	2	33.3	
	7 × 10^6^	4	66.7	
	9.5 × 10^6^	6	100	
WH01-3A	2.5 × 10^8^	0	0	7.4 × 10^8^
	4.5 × 10^8^	0	0	
	6.5 × 10^8^	0	0	
	7.5 × 10^8^	3	50	
	1 × 10^9^	6	100	
NC	PBS	0	0	

### Immune protection in mice

Seventy-two 6-week-old female BALB/c mice were randomly divided into nine groups (*n* = 8). Each group was inoculated with either vaccine or PBS via intramuscular injection. Groups 1–3 were immunized with 50 μL of 2 × 10^8^ CFU of the WH01-3A mutant. Groups 4–6 were immunized with 50 μL of the commercial subunit vaccine (Porcilis^®^ APP, Merck & Co, USA), containing three inactivated exotoxins (ApxI, ApxII, ApxIII) and a 42-kDa outer membrane protein (OMP). This vaccine was chosen as the comparator because it includes all three exotoxins and provides the broadest serotype coverage currently available (serovars 1–15). In addition, our engineered vaccine strain is likewise capable of simultaneously expressing these three exotoxins. Groups 7–9 were injected with 50 μL of PBS. At 14 days after the primary immunization, each group received a booster of the same dose. Then, 14 days following the booster, groups 1, 4, and 7 were challenged with 200 μL of 2.3 × 10^7^ CFU of *A. pleuropneumoniae* serovar 1 strain WH01. Groups 2, 5, and 8 were challenged with 200 μL of 1.7 × 10^7^ CFU of *A. pleuropneumoniae* serovar 5 strain 202203179. Groups 3, 6, and 9 were challenged with 200 μL of 1.2 × 10^8^ CFU of *A. pleuropneumoniae* serovar 15 strain XB2T-56. All challenges were performed via intraperitoneal injection. Survival rates were recorded 72 h post-challenge. During the immunization period, serum samples from each group were collected to assess antibodies against whole-bacterial cells of serovars 1 (WH01), 5 (202203179), and 15 (XB2T-56), as well as the ApxIA, ApxIIA, and ApxIIIA proteins.

### Safety assessment in pigs

Twelve 40-day-old pigs were randomly divided into three groups (*n* = 4). On day 0, Groups 1, 2, and 3, received 1 mL of 2 × 10^7^ CFU (low-dose), 1 × 10^8^ CFU (middle-dose), and 5 × 10^8^ CFU (high-dose) of the WH01-3A mutant via intramuscular injection. On days 3, 7, and 10, nasal and tonsil swabs were collected from each pig and placed in 1 mL of physiological saline, and mixed by vortexing. Subsequently, 50 μL of the mixtures were evenly spread onto TSA plates. All resulting bacterial colonies were collected, and genomic DNA was extracted to be used as template for PCR using primers P*nap*-F/*apxIIIA*-R. On day 14, each group received a second injection at the same dose. Following inoculation, body temperature and clinical symptoms were monitored daily. On day 42, pigs in the low-dose and high-dose groups were necropsied to examine lung and tonsil lesions and for CFU determinations in tissue samples. Serum samples from the middle-dose group were collected on days 1 (pre-immunization), 14, 28, 42, 56, 70, and 81 for anti-Apx toxin ELISA antibody titer.

### WH01-3A immune protection in pigs

Twenty-seven 55-day-old pigs were randomly divided into five groups. Groups 1 and 2 (*n* = 6) received 1 mL of 1 × 10^8^ CFU of the WH01-3A mutant via the intramuscular route. Groups 3 and 4 (*n* = 6) received 1 mL of PBS via the same route. Group 5 was the blank control group (*n* = 3). All groups received a booster immunization with the same dose on day 30, and serum samples were collected on days 28 and 60 for antibody detection. On day 75, groups 1 and 3 were intranasally challenged with 1 mL of *A. pleuropneumoniae* serovar 1 strain WH01 at 1.2 × 10^8^ CFU, while groups 2 and 4 were intranasally challenged with 1 mL of *A. pleuropneumoniae* clinical serovar 15 strain XB2T-56 at 6 × 10^8^ CFU. Body temperature and clinical symptoms were monitored daily. On day 9 post-challenge, all surviving pigs were necropsied. Clinical symptoms were scored on a scale of 0–4 according to severity, with specific criteria referenced from [38]. In this study, lung lesions following *A. pleuropneumoniae* infection were evaluated using the Consolidation Lung Lesion Score (LLS) system proposed by Hannan et al. [39]. Each lung lobe is divided into several triangles (7 for each pair of cranial and middle lobes, 19 for each pair of diaphragmatic lobes, and 8 for the accessory lobe). The number of lesion-affected triangles in each lobe is multiplied by five and divided by the total number of triangles in that lobe, so that a lobe completely affected by lesions receives a score of 5. The maximum total score is 35 [40]. Lung lesion scoring was performed by the same investigator, who identified samples only by their coded numbers and was unaware of the group allocation during scoring. Lung tissues were fixed in formalin after collection and sent to Wuhan Baiqiandu Technology Co., Ltd. for preparation of tissue sections and hematoxylin–eosin (H&E) staining and analysis. During submission, only sample identification numbers were provided, and no information regarding group allocation was disclosed.

Lung and tonsil lesion samples were inoculated onto TSA plates, and incubated at 37 °C for 12 h. All bacterial colonies were collected, their genomic DNA extracted for use as template in PCRs. The presence of *A. pleuropneumoniae* was first confirmed using *apxIVA*-F/R primers, and then serovar-specific primers AP1F/R and AP15F/R, for serovar 1 and 15, respectively.

### Antibody tests

Serum antibody levels or titers were determined using indirect ELISAs. Briefly, 100 μL of 5 μg/mL WH01 antigen (or 202203179, XB2T-56, ApxIA, ApxIIA, ApxIIIA) diluted in 50 mM carbonate buffer (pH 9.6), was added to 96-well plates and incubated overnight at 4 °C. Wells were washed three times with 300 μL PBST buffer (PBS supplemented with 0.05% Tween-20), and then blocked with 300 μL PBST containing 5% skim milk at 37 °C for 1 h, followed by three additional washes with PBST. Subsequently, 100 μL of serum samples diluted 1:160 in PBST (or serially diluted sera for titer determination) were added to the wells and incubated at 37 °C for 1 h. After washing the plates, 100 μL of HRP-conjugated goat anti-mouse IgG (or goat anti-swine IgG) (Proteintech, Wuhan, China) diluted 1:5000 was added to each well and incubated at 37 °C for 1 h. After three washes, 100 μL of tetramethylbenzidine (TMB) substrate solution (Beyotime, Shanghai, China) was added and incubated for 20 min. The OD_630_ was measured using a microplate reader (Multiskan™ FC, Thermo, USA). To evaluate the DIVA-related characteristics of the vaccine strain, serum samples collected from immunized pigs at 28 and 60 days after primary immunization were tested using a commercial ApxIVA ELISA kit (JNT, Beijing, China). The assay was performed as described in the “Animal experiments” section.

### Statistical analysis

All data analyses and graphical representations were performed using GraphPad Prism^®^ 8.0 software. Quantitative data are presented as the mean ± standard deviation (SD). For the in vitro biofilm formation assay, the OD values obtained from each group were analyzed using one-way analysis of variance (ANOVA), followed by Tukey’s post hoc test for pairwise comparisons when the overall difference was statistically significant. For the levels of antigen-specific IgG in mouse and pig sera, the OD values of serum samples from different groups were analyzed using two-way ANOVA, with treatment group and sampling time point as factors; when the overall difference was statistically significant, multiple comparisons were performed using Tukey’s post hoc test. A *p* value < 0.05 was considered statistically significant. Statistical significance in the figures is indicated as follows: *, *p* < 0.05; **, *p* < 0.01; ***, *p* < 0.001.

## Results

### Construction and characterization of the attenuate mutant WH01-3A

**Figure 1:**
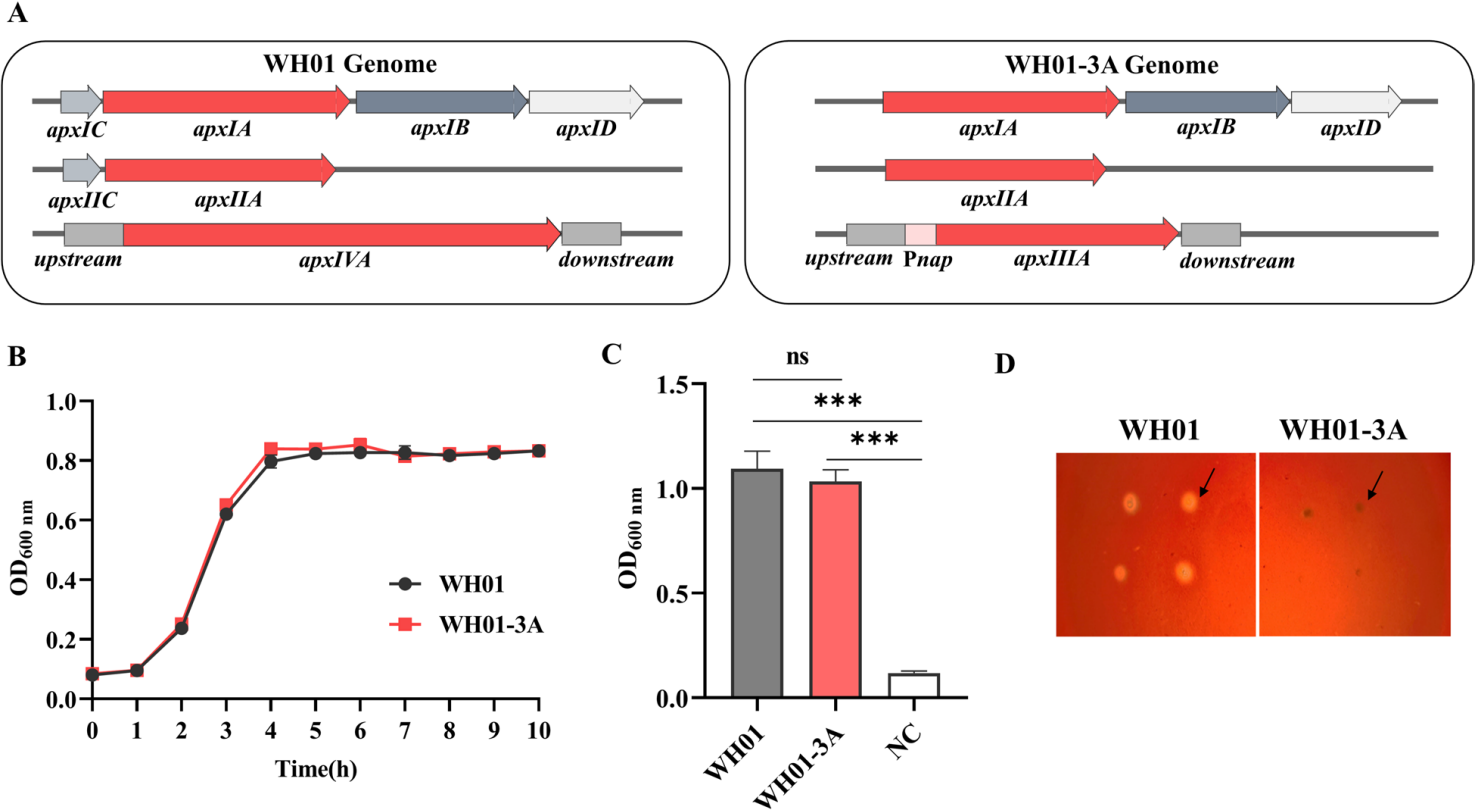
**Construction and biological characterization of the attenuated mutant strain WH01-3A.** **A** Schematic representation of the relevant *apx* genomic loci of WH01 and the WH01-3A mutant. **B** In vitro growth curves of WH01 and the WH01-3A mutant. Overnight cultures were inoculated into fresh TSB medium containing NAD and incubated at 37°C with shaking. OD_600 nm_ values were determined hourly OD_600 nm_. Data are presented as means ± SD from three independent replicates. **C** In vitro biofilm formation of the WH01 and the WH01-3A mutant. Overnight cultures were inoculated into fresh BHI medium containing NAD and statically incubated at 37°C. NC: BHI medium. After 5 h, the supernatant in the wells was discarded, and biofilm formation quantitatively assessed using 0.1% crystal violet ammonium oxalate solution. Data are presented as means ± SD from three independent replicates. Data analysis was performed using one-way ANOVA (∗ , *p* < 0.05; ∗  ∗ , *p* < 0.01; ∗  ∗  ∗ , *p* < 0.001; ns, not significant). **D** Hemolytic activity of WH01 and the WH01-3A mutant. Both strains were inoculated onto TSA plates containing NAD and 5% defibrinated sheep erythrocytes, and after static incubation at 37°C for 16 h, hemolytic rings around the colonies were observed for WH01 but not WH01-3A. The black arrow indicates the location of one of the bacterial colonies.

*A. pleuropneumoniae* serovar 1 WH01 was used as the parental strain, which was attenuated through sequential deletions of its *apxIVA*, *apxIC*, and *apxIIC* genes. Subsequently, the *apxIIIA* gene with the promoter region of the *napFDAGHBC* operon, encoding nitrate reductase (*nap* promoter) [31], was inserted at the *apxIVA* locus of the triple deletion mutant, resulting in mutant WH01-3A (Figure 1A and Additional file 2A). The gene deletions and insertions were confirmed by PCR using genomic DNA from WH01-3A and WH01 (control) as templates (Additional file 2A). The *apxIIIA* gene was successfully inserted into the WH01-3A mutant genome (Additional file 2B). The growth rates of WH01-3A and WH01 were comparable (Figure 1B), indicating that the gene deletions and insertions had no impact on bacterial growth. The WH01-3A mutant retained normal biofilm formation under static in vitro conditions (Figure 1C). Notably, in contrast to WH01, WH01-3A colonies were not surrounded by hemolytic rings (Figure 1D). In a western blot, the native toxin protein purified from the WH01-3A supernatant exhibited specific reactivity with mouse antiserum against ApxIA, ApxIIA, and ApxIIIA. (Additional file 2C). Additionally, all introduced genetic modifications in the WH01-3A mutant were stably inherited after 20 consecutive passages (Additional file 2D).

### WH01-3A is less virulent than WH01 in mice

The comparative virulence of WH01-3A and WH01 was determined in mice. Mortalities after infection with different doses of WH01-3A and WH01 are shown in Table 1. Using the Reed-Muench method, the LD_50_s of WH01 and WH01-3A were calculated to be 4.9 × 10^6^ CFU and 7.4 × 10^8^ CFU, respectively. Thus, compared with WH01, the WH01-3A mutant was approximately 150-fold less virulent in mice.

### A WH01-3A live attenuated vaccine protects mice against challenge with *A. pleuropneumoniae* serovars 1, 5, and 15

**Figure 2:**
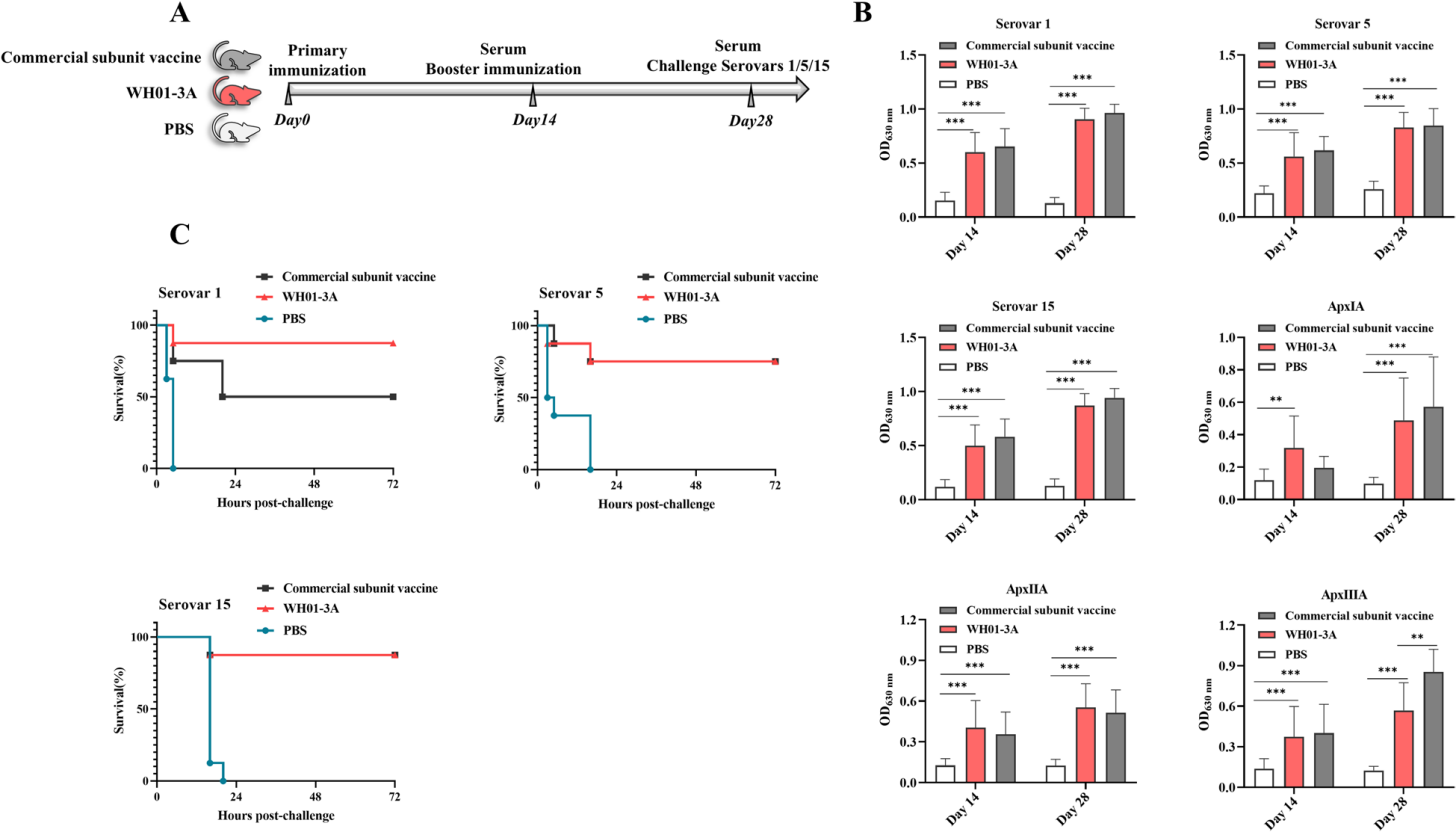
**WH01-3A vaccination results in protection of mice against A. pleuropneumoniae serovars 1, 5, and 15 challenge.** **A** Vaccination and challenge process on BALB/c mouse. On day 0, mice were immunized intramuscularly with 50 μL of either commercial subunit vaccine or WH01-3A (2 × 10^8^ CFU), followed by a booster immunization on day 14. Sera were collected on days 14 and 28 for antibody detection by ELISA experiments. On day 28, mice were challenged with either 2.3 × 10^7^ CFU (200 μL) of *A. pleuropneumoniae* serovar 1 strain WH01, 1.7 × 10^7^ CFU (200 μL), serovar 5 strain 202203179, or 1.2 × 10^8^ CFU (200 μL) serovar 15 strain XB2T-56. At 72 h post-challenge, the percentage survival for each group was calculated. **B** Levels of specific IgG antibodies in mouse sera from each group on days 14 and 28 were detected by indirect ELISA, using serovar 1 strain WH01, serovar 5 strain 202203179, serovar 15 strain XB2T-56, ApxIA, ApxIIA, and ApxIIIA as the coating antigens. Data are presented as means ± SD from 24 mice in each group. Data analysis was performed using two-way ANOVA (∗ , *p* < 0.05; ∗  ∗ , *p* < 0.01; ∗  ∗  ∗ , *p* < 0.001; ns, not significant). **C** Percentage survival of each group up to 72 h post-challenge with serovar 1 strain WH01, serovar 5 strain 202203179, and serovar 15 strain XB2T-56.

The efficacy of the WH01-3A mutant as a live attenuated vaccine in preventing *A. pleuropneumoniae* serovar 1 (homologous) and serovars 5, and 15 (heterologous) infection was evaluated in BALB/c mice (Figure 2A). A commercial subunit vaccine (Porcilis^®^ APP) was used as a control.

After booster immunization, antibodies against various antigens increased in all immunized groups compared with those observed 14 days after the primary immunization (Figure 2B). Both the WH01-3A group and the commercial subunit vaccine group exhibited significantly higher antibody levels against tested antigens than the PBS control group (*p* < 0.001) (Figure 2B). For antibodies against serovars 1, 5, 15, ApxIA, and ApxIIA, no significant differences were observed between the two immunized groups. The commercial subunit vaccine group exhibited higher antibody levels against ApxIIIA than the WH01-3A group (*p* < 0.01) (Figure 2B).

After booster immunization, challenge experiments were conducted using clinical isolates of *A. pleuropneumoniae* serovars 1, 5, and 15. All mice in the PBS control group died within 24 h, while those in the immunized groups exhibited mild clinical symptoms within 2 h post-challenge, e.g., ruffled fur and decreased appetite. Upon challenge with the parental serovar 1 strain WH01, 87.5% of the WH01-3A immunization group were protected, which was higher than the 50% protection in the commercial vaccine group (Figure 2C). Against heterologous serovars, WH01-3A and the commercial vaccine protected 75% and 87.5% of mice against serovars 5 and 15 challenge, respectively (Figure 2C). These results indicate that both the WH01-3A live attenuated vaccine and the commercial subunit vaccine can provide cross-protection against the serovars investigated. These findings highlight the broad-spectrum potential of the WH01-3A mutant as a live attenuated *A. pleuropneumoniae* vaccine in mice models and providing a basis for further studies in host animals.

### WH01-3A is safe for pigs

**Figure 3:**
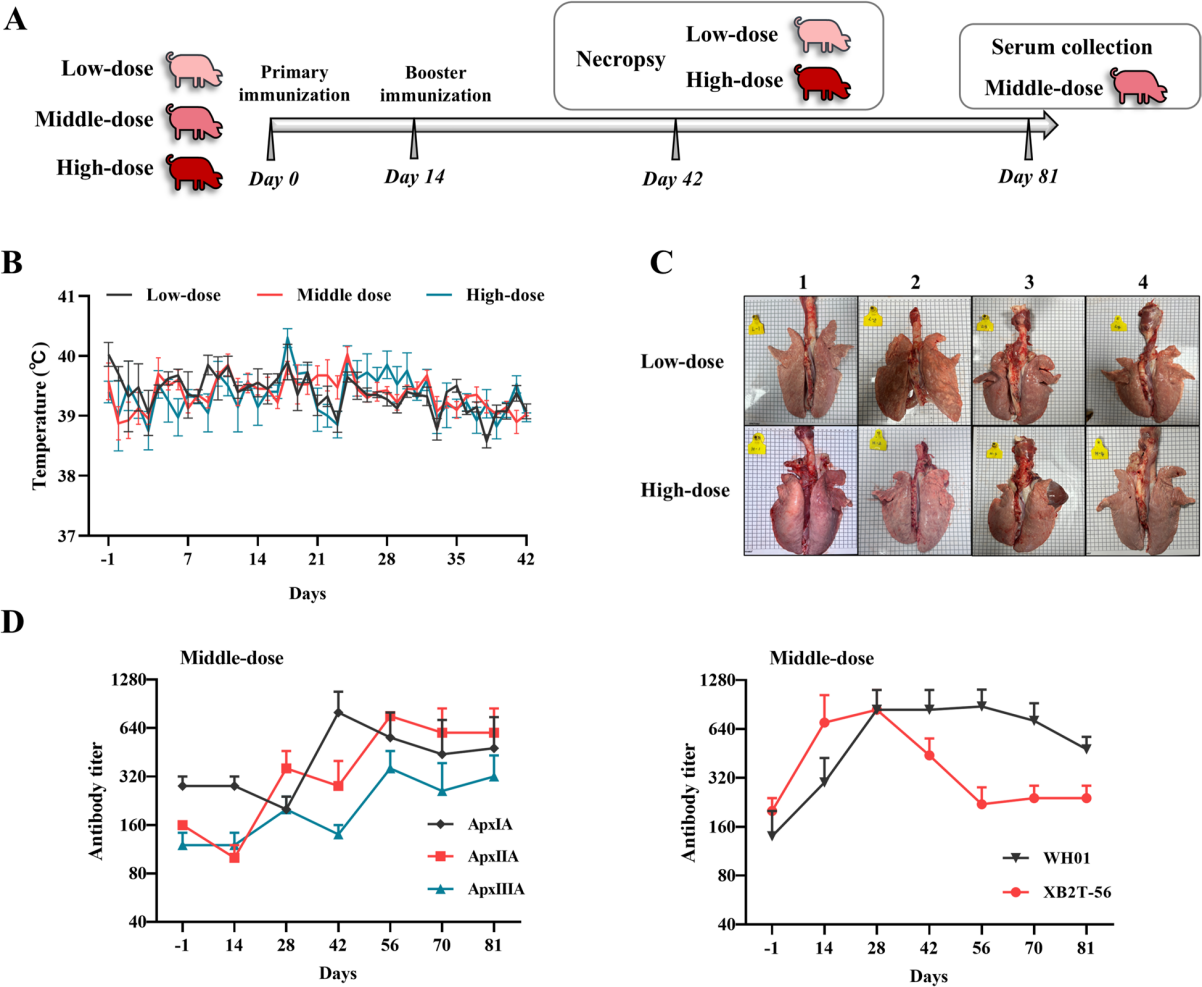
**Safety assessment of WH01-3A in pigs.** **A** Schematic showing the protocol for evaluating the safety of WH01-3A in pigs. Forty-day-old pigs were intramuscularly injected with three different doses of the WH01-3A mutant: 2 × 10^7^ CFU (Low-dose, *n* = 4), 1 × 10^8^ CFU (Middle-dose, *n* = 4), and 5 × 10^8^ CFU (High-dose, *n* = 4). Primary immunization was on day 0, followed by a booster on day 14. On day 42, the low-dose and high-dose groups were necropsied, while antibody titers in the serum of the middle-dose group were continuously monitored until day 81. **B** Body temperatures of pigs in the three immunization groups were recorded daily. Data are presented as means ± SD for all pigs in each group. **C** Comparison of lungs from pigs in the low- and high- dose groups after euthanasia on day 42. **D** Serum antibody titers in the middle-dose group during immunization, assessed using serovar 1 strain WH01, serovar 15 strain XB2T-56, ApxIA, ApxIIA, and ApxIIIA as coating antigens. Data are presented as means ± SD for all pigs in middle-dose group.

The safety of the WH01-3A as a live attenuated vaccine in pigs was evaluated by assessing clinical symptoms, potential for transmission, and lung and tonsil pathology of inoculated pigs. Three groups of 40-day-old pigs were intramuscularly immunized with different doses of WH01-3A, designated as low-dose, medium-dose, and high-dose groups (Figure 3A). During the observation period after both the primary and booster immunizations, all groups exhibited normal feed intake and mental status. The mean body temperature fluctuated between 39 and 40 °C. On day 17, one pig in the high-dose group exhibited an increase in body temperature to 40.6 °C, which subsequently returned to normal immediately afterward (Figure 3B). PCR analysis of tonsil and nasal swabs collected within 10 days after the primary immunization failed to detect WH01-3A (Additional file 3A-F). On day 42, necropsies of the low-dose and high-dose groups revealed no pleuropneumonia-associated pathological changes in the lungs or tonsils (Figure 3C). PCR did not detect WH01-3A in lungs or tonsils (Additional file 4A-B). These results indicate that WH01-3A may not have established detectable colonization in pigs when administered at doses ranging from 2 × 10^7^ to 5 × 10^8^ CFU. Immune sera from the middle-dose group were collected until day 81 post-immunization. Compared to pre-immunization levels, antibody titers against whole-cell proteins of serovars 1 and 15 peaked at day 28. Thereafter, anti-serovar 15 antibodies began to fluctuate and then decrease. Conversely, serovar 1 antibody titers were maintained until a decrease at day 56 post-immunization (Figure 3D). Antibody titers against ApxIA peaked at day 42 post-immunization and then decreased, ApxIIA and ApxIIIA titers peaked at day 56 post-immunization before starting to decrease (Figure 3D).

### WH01-3A immunization prevents infection in pigs challenged with *A. pleuropneumoniae* serovars 1 and 15

**Figure 4:**
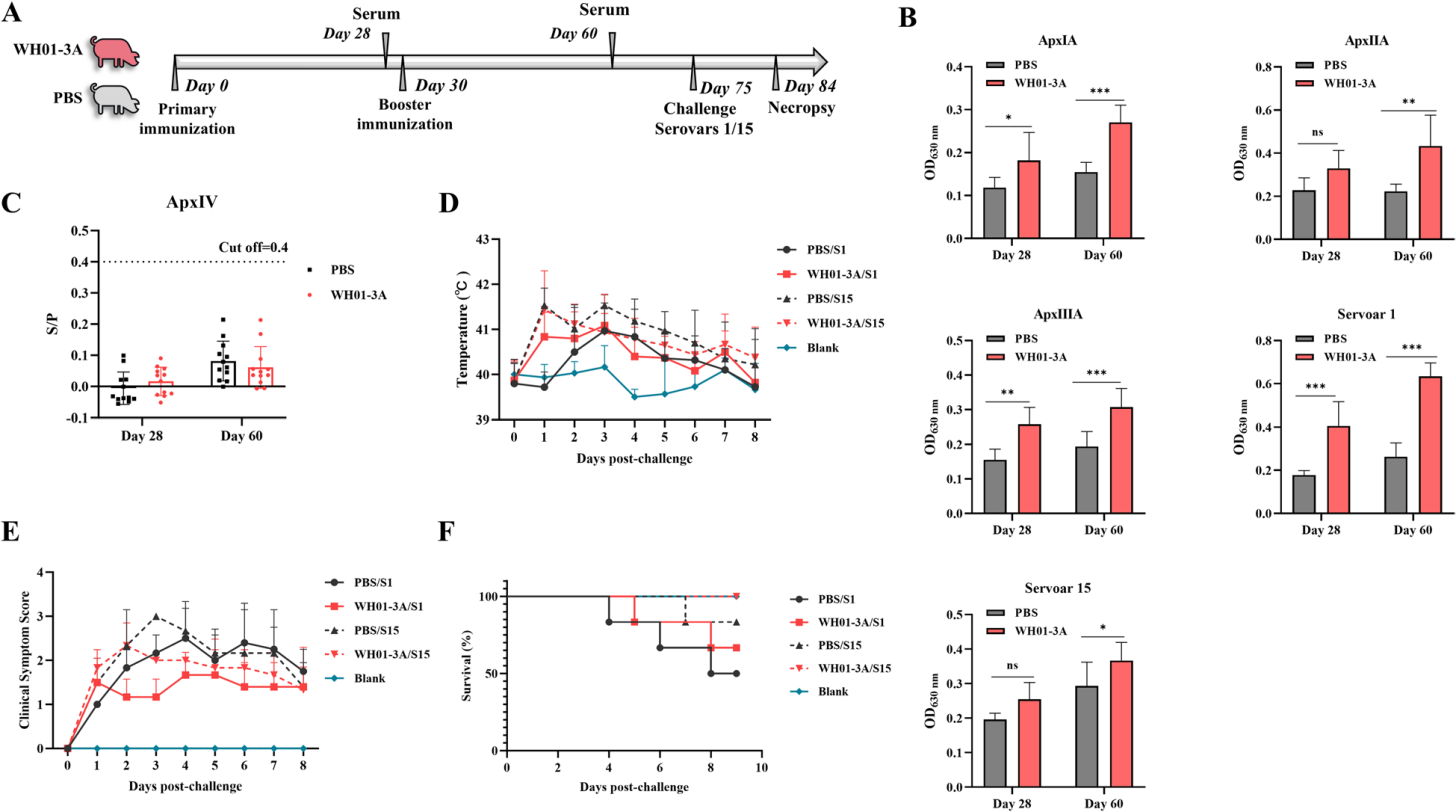
**Immune protection of WH01-3A in pigs.** **A** Schematic of pig vaccination and challenge schedule. On day 0, each group was injected intramuscularly with 1 mL bacteria containing 1 × 10^8^ CFU of WH01-3A (*n* = 6) or 1 mL of PBS (*n* = 6), with a blank control group (*n* = 3). A booster immunization was administered on day 30. Serum samples were collected on days 28 and 60 for antibody detection by ELISA. On day 75, pigs were challenged intranasally with either 1 mL bacteria containing 1.2 × 10^8^ CFU of *A. pleuropneumoniae* serovar 1 strain WH01 (*n* = 6) or 6 × 10^8^ CFU of serovar 15 strain XB2T-56 (*n* = 6). On the ninth day post challenge, all surviving pigs were necropsied. **B** Specific IgG antibody levels in the serum of each group on days 28 and 60 were measured by indirect ELISA, using serovar 1 strain WH01, serovar 15 strain XB2T-56, ApxIA, ApxIIA, and ApxIIIA as coating antigens. Data are presented as means ± SD from all the pigs in each group. Data analysis was performed using two-way ANOVA (∗ , *p* < 0.05; ∗  ∗ , *p* < 0.01; ∗  ∗  ∗ , *p* < 0.001; ns, not significant). **C** The levels of ApxIVA antibodies in the serum of pigs from each group on days 28 and 60 were measured using an ApxIVA ELISA kit. Data are presented as means ± SD for all pigs in each group. Note: When the sample OD value is lower than that of the negative control, the S/P result is negative. **D**, **E** Changes in body temperature (**D**), clinical symptoms (**E**), and percentage survival (**F**) following challenges with serovar 1 strain WH01 and serovar 15 strain XB2T-56 after vaccination with WH01-3A or PBS. Body temperature and clinical symptoms data are presented as mean ± SD from all pigs in each group.

Finally, we evaluated the protective efficacy of the WH01-3A mutant as a live attenuated vaccine against challenge with A. *pleuropneumoniae* serovars 1 and 15 in pigs. Fifty-five-day-old pigs were used for the primary immunization, followed by a booster at day 30. On day 75 post-immunization, pigs were challenged with serovar 1 WH01 or serovar 15 strain XB2T-56. At 9 days after challenge, surviving pigs were necropsied (Figure 4A). ELISA results after primary and booster immunizations showed that the WH01-3A immunized group had high levels of specific antibodies against the toxin proteins ApxIA, ApxIIA, ApxIIIA, as well as against whole-cell proteins of serovars 1 and 15 (*p* < 0.05) (Figure 4B). Moreover, no ApxIVA antibodies were detected (Figure 4C).

**Figure 5:**
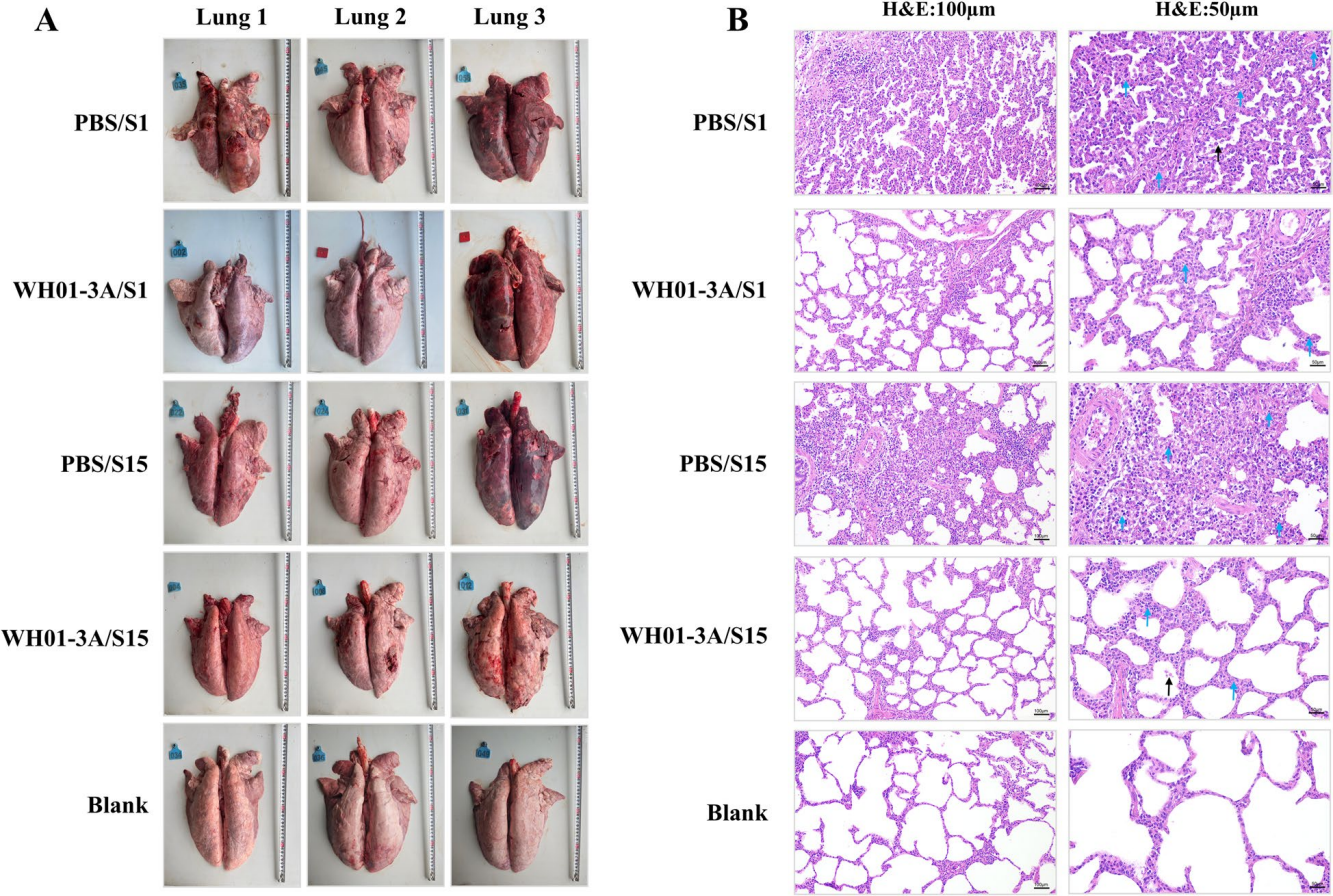
**Histopathological and lung lesion analyses of pigs.** **A** Comparison of the lung lesion severity among groups. Lungs showing the typical lesions from three pigs in each group are shown. **B** Representative sections of lung tissue were stained with hematoxylin and eosin (H&E). Photomicrographs were obtained at magnifications corresponding to scales of 100 μm and 50 μm for pathological analysis. The blue arrows indicate localized thickening of the alveolar walls, accompanied by proliferation of alveolar epithelial cells. The black arrows indicate atrophy of the alveolar cavity structure, with a small number of exfoliated cells present within the cavity.

Upon challenge with serovar 1, the body temperature of both the WH01-3A and PBS groups peaked on the third day post-challenge and gradually returned to normal. The WH01-3A group exhibited milder clinical symptoms than the PBS group overall (Figure 4D, E). Following challenge with serovar 15, body temperature in both groups peaked on the first day post-challenge and then decreased to normal, with the WH01-3A group presenting milder clinical symptoms (Figure 4D, E). Survival rates prior to euthanasia were 66.7% and 100% in the WH01-3A group for serovar 1 and 15 challenge, respectively, compared with 50% and 83.3% in the PBS group (Figure 4F). The mean lung lesion score in the PBS group challenged with serovar 1 was 23.396, compared with 13.421 in the WH01-3A group, a 42.6% reduction. For serovar 15 challenge, the mean lung lesion score was 8.415 in the PBS group and 2.707 in the WH01-3A group, a 67.8% reduction (Table 2). After challenge with serovar 1, lung and tonsil bacterial isolation rates were both 100% in the PBS group, and 100% and 83.3%, respectively, in the WH01-3A group. For serovar 15, the lung and tonsil bacterial isolation rates were both 100% in the PBS group, and 66.7% and 33.3% in the WH01-3A group, respectively (Table 2). Histopathological examination identified severe lung lesions in the PBS group after challenge with either serovars 1 or 15. Comparatively mild lesions were found in the WH01-3A immunized and challenged group (Figure 5). No lesions were found in the blank (PBS, not challenged) group. Overall, WH01-3A provided immunological protection and reduced the extent of lung lesions in pigs after *A. pleuropneumoniae* homologous serovar 1 and heterologous serovar 15 challenge, indicating its potential as an effective candidate vaccine strain.

**Table 2:** **Lung lesion scores, lung and tonsil bacterial isolation for each pig after* A. pleuropneumoniae*** **infection**

Group	ID	Lung lesion score^a^	Average score^b^	Reduction %^c^	Lung bacterial isolation	Lung bacterial isolation (%)	Tonsil bacterial isolation	Tonsil bacterial isolation (%)
PBS/S1 (*n* = 6)	030	35.00	23.396	/	+	100%	+	100%
	035	22.86			+		+	
	045	3.27			+		+	
	051	35.00			+		+	
	052	9.25			+		+	
	058	35.00			+		+	
WH01-3A/S1 (*n* = 6)	001	35.00	13.421	42.6%	+	100%	–	83.3%
	002	1.58			+		+	
	005	1.05			+		+	
	009	2.37			+		+	
	011	35.00			+		+	
	016	5.53			+		+	
PBS/S15 (*n* = 6)	022	3.16	8.415	/	+	100%	+	100%
	023	7.11			+		+	
	024	2.74			+		+	
	026	1.05			+		+	
	031	35.00			+		+	
	055	1.43			+		+	
WH01-3A/S15 (*n* = 6)	004	0.53	2.707	67.8%	+	66.7%	–	33.3%
	008	2.63			+		–	
	012	7.07			+		+	
	013	1.58			–		+	
	018	1.05			–		–	
	020	3.38			+		–	
Blank (*n* = 3)	034	0	0	/	–	0	–	0
	036	0			–		–	
	049	0			–		–	

a: Lung lesions in each pig from each group were scored individually using the LLS method [40].

b: Mean lung lesion scores for each group of pigs.

c: Percentage reduction of mean lung lesion score in the WH01-3A group compared with the PBS group for the same challenge strain.

## Discussion

Vaccination is one of the primary strategies for controlling *A. pleuropneumoniae* infection. However, the lack of cross-protection among different serovars limits its effectiveness [16]. *A. pleuropneumoniae* live attenuated vaccines can elicit comprehensive immune responses, and potentially protect against different serovars [15]. The *A. pleuropneumoniae* ApxI-ApxIII toxins are both key virulence factors and vaccine components [17]. Previous studies have constructed a serovar 10 *apxIA* mutant that produces a C-terminally truncated ApxI toxin by insertion of a chloramphenicol resistance gene cassette [28]. However, the presence of the antibiotic resistance gene limits its practical application. Another study deleted the *C* genes of the ApxI and ApxII toxins in a serovar 1 strain, eliminating virulence while retaining the immunogenicity of ApxIA and ApxIIA [41]. However, such conventional attenuated strains cannot differentiate between naturally infected and vaccinated animals [15]. Liu et al. [42] constructed a serovar 7 Δ*apxIIC*Δ*apxIVA* mutant by deleting 605 bp from the N-terminus of the *apxIVA* gene, enabling serological differentiation between naturally infected and vaccinated animals. However, serovar 7 strains express only the ApxII toxin, limiting vaccine use. On the basis of these strategies, the present study aimed to develop a novel live attenuated vaccine with broader protection that can differentiate between naturally infected and vaccinated animals. We selected the virulent serovar 1 clinical isolate WH01 as the parental strain, deleting the *apxIVA*, *apxIC*, and *apxIIC* genes to achieve sufficient attenuation while retaining ApxIA and ApxIIA immunogenicity. This approach allows the vaccine strain to be readily distinguished in serological diagnostics. To broaden the protective spectrum, a heterologous *apxIIIA* gene was subsequently introduced into the deleted *apxIVA* locus, resulting in mutant WH01-3A, which simultaneously expresses ApxIA, ApxIIA, and ApxIIIA. To ensure effective expression of the exogenous gene, the strong promoter P*nap* [31] was introduced upstream of *apxIIIA* (Figure 1A). Unlike WH01, WH01-3A was nonhemolytic (Figure 1D) and approximately 150-fold less virulent than the parental strain in mice (Table 1). We attribute WH01-3A attenuation to the deletion of the virulence genes *apxIVA*, *apxIC*, and *apxIIC*. Western blotting confirmed that ApxIA, ApxIIA, and ApxIIIA was expressed in WH01-3A (Additional file 2C). Furthermore, immunization of both mice and pigs induced serum antibodies against ApxIA, ApxIIA, and ApxIIIA (Figure 2B and 4B).

In addition, because the vaccine strain lacks the *apxIVA* gene, no ApxIVA-specific antibodies were detected during the immunization observation period in this study, indicating that this vaccine has the potential to differentiate naturally infected animals from vaccinated animals. Immunized pigs can be tested at different time points after vaccination, such as at slaughter or during routine surveillance, using a commercial ApxIVA ELISA kit. Vaccinated-only pigs are expected to be negative, whereas a positive result indicates field strain infection or exposure. It should be noted that the sensitivity and specificity of the DIVA assay are related to the sampling time and the time of infection. Although the ApxIV ELISA has relatively high specificity, its sensitivity is comparatively limited. Therefore, its DIVA effect may be constrained in herds with subclinical infection, in pigs carrying *A. pleuropneumoniae* only in the tonsils, or in animals infected with strains containing genomic insertion sequences that may block ApxIV toxin production. In addition, this assay cannot distinguish vaccinated pigs that were subsequently infected from unvaccinated infected pigs. Therefore, its practical use should be combined with clinical observation for regular herd-level surveillance.

In safety evaluations, WH01-3A did not cause any observable clinical symptoms of pleuropneumonia in pigs. In addition, WH01-3A was not detected in nasal or tonsillar swabs collected at the predetermined sampling time points. In further studies, co-housed sentinel pigs can be included for transmission assessment following immunization.

The Porcilis^®^ APP is a commercial vaccine intended for pigs and can be used for broad-spectrum immunization against multiple *A. pleuropneumoniae* serovars. According to the recommended immunization dose for pigs and allometric conversion based on body surface area [43], the theoretical equivalent injection volume for a mouse weighing approximately 20 g was estimated to be about 15–20 μL. However, as the candidate vaccine in this study is a live attenuated vaccine, the use of an excessively small intramuscular injection volume would require a higher bacterial concentration, which may increase dosing variability and adversely affect local tolerability. Therefore, to ensure consistency of administration conditions across groups and the reproducibility of the immunization procedure, a uniform injection volume of 50 μL per mouse was used in this study. In addition, preliminary experiments confirmed that the commercial vaccine was safe in mice at this injection volume.

The production of antigen-specific IgG antibodies following vaccination is important for protection against infection. For *A. pleuropneumoniae*, neutralizing antibodies against Apx toxins can protect neutrophils from being killed, thereby maintaining the host’s capacity to clear bacteria [15, 44, 45]. Our data shows that WH01-3A can elicit a humoral immune response. In the mouse model, immunization with WH01-3A induced not only serovar 1-specific IgG antibodies but also cross-reactive antibodies against serovars 5 and 15. In addition, specific IgG antibodies against the three key antigens ApxIA, ApxIIA, and ApxIIIA were all detected in sera (Figure 2B).

Although the IgG antibody level against ApxIIIA was lower than that induced by the commercial subunit vaccine, mice immunized with WH01-3A had a comparable protective efficacy to the commercial vaccine when challenged with a serovar 15 strain (Figure 2C). These results suggest that the immune protection conferred by WH01-3A does not rely solely on antibodies against the three toxins. In the pig model, the efficacy of WH01-3A were further validated. Serum IgG antibody levels against all three Apx toxins in immunized pigs were significantly higher than those in the PBS control group (Figure 4B). Correspondingly, in challenge protection assays, the survival rates of the WH01-3A immunized group were higher than those of the PBS control group, regardless of challenge with homologous serovar 1 or heterologous serovar 15 strains (Figure 4F). These findings indicate that this vaccine candidate is able to provide effective immunological protection in host animals.

After intramuscular immunization with WH01-3A, the strain was not detected at the timepoints analyzed in the nasal cavity, tonsils, or lung tissue, substantially reducing the risk of environmental shedding. Despite the apparent absence of tissue colonization, antibodies against ApxIA, ApxIIA, ApxIIIA, and whole bacteria were detected in the sera of immunized animals (Figure 3D and 4B), providing excellent immune protection (Figure 4F and 5A). It is generally believed that intranasal vaccination can elicit a strong mucosal immune response, including the production of secretory IgA [46]. However, previous studies have shown that intramuscular immunization with attenuated *A. pleuropneumoniae* vaccines induces better immunogenicity than intranasal administration [47]. One possible explanation is that effective clearance of encapsulated pathogens, such as *A. pleuropneumoniae*, depends on robust opsonophagocytic activity mediated by systemic IgG and complement activation. Specific IgG directed against surface polysaccharides and secreted toxins, such as ApxI/II/III, plays a crucial role in neutralizing virulence factors and promoting bacterial clearance. The enhanced protective efficacy observed following intramuscular immunization with attenuated *A. pleuropneumoniae* vaccines may therefore be attributed to its ability to rapidly induce systemic IgG and IFN-γ responses, both of which are essential for opsonization, phagocytosis, and Th1-mediated bacterial elimination [47].

It was observed that the increase in antibody titer against ApxIIIA was lower than that against ApxIA and ApxIIA. Although not investigated further in this study, we hypothesize that secretion of all three Apx toxins in the WH01-3A strain depends on the ApxIBD transporter system and is subject to “transport stress,” resulting in different secretion efficiencies. No natural isolate of *A. pleuropneumoniae* has been identified that expresses ApxIA, ApxIIA, and ApxIIA, indicating that this is burdensome to the bacterium. Otherwise, it might have been expected to have occurred during evolution of the bacterium. Our attempts to integrate a complete ApxIIIABD gene cluster into the genome to optimize ApxIIIA secretion were unsuccessful. Therefore, future research will aim to optimize the efficiency of the ApxIBD transporter to enhance the expression and secretion of all three Apx toxins and improve the efficacy of the vaccine.

In summary, we constructed a live attenuated *A. pleuropneumoniae* WH01-3A vaccine strain expressing ApxIA, ApxIIA, ApxIIIA, but not ApxIVA, which protected mice and pigs against homologous and heterologous serovars.

## Supplementary Information

**Additional file 1. Bacterial strains, plasmids and primers used in this study.**

**Additional file 2. Identification of the WH01-3A strain.** (**A**). Genomic DNA was extracted from WH01-3A and WH01. Primers Δ*apxIV*-F/R amplified an internal fragment of the *apxIV* gene. Primers *apxIV*-UF/DR amplified the upstream and downstream regions of *apxIV*. Primers P*nap*-F/*apxIIIA*-R amplified an internal fragment of the inserted gene. Primers Δ*apxIC*-F/R amplified an internal fragment of the *apxIC* operon. Primers *apxIIC*-UF/DR amplified upstream and downstream regions of *apxIIC*. (**B**). RNA was extracted from A. pleuropneumoniae JL03, Δ*apxIV*Δ*apxIC*Δ*apxIIC*, and WH01-3A , and cDNA was synthesized by RT-PCR after gDNA removal. PCR identification with the primer *apxIIIA*-F/*apxIIIA*-R. (**C**). The expression of native toxins non-acylated ApxIA, ApxIIA, and ApxIIIA in the WH01-3A supernatant was analyzed by western blotting. Anti-ApxIA, ApxIIA, or ApxIIIA mouse serum were used separately as primary antibodies, with the corresponding recombinant proteins rApxIA, rApxIIA, or rApxIIIA serving as positive controls. To verify the specificity of the detection method, A. pleuropneumoniae strains with distinct toxin secretion profiles were tested in parallel: serovar 1 strain WH01 secretes acylated ApxIA, ApxIIA proteins, serovar 7 strain AP76 secretes only acylated ApxIIA protein, and serovar 10 strain D13039 secretes only acylated ApxIA protein. Following SDS-PAGE and transfer to a PVDF membrane, proteins were probed with Anti-ApxIA, ApxIIA, or ApxIIIA mouse serum (1:5000, 4°C, overnight) and subsequently with HRP-conjugated goat anti-mouse IgG (1:10,000, 37°C, 1 h). Detection was performed by enhanced chemiluminescence. (**D**). Genetic stability of the knockout or inserted genes during serial passage. Genomic DNA was extracted from even-numbered (2-20) passages of WH01-3A, with WH01 genomic DNA used as a control.

**Additional file 3. PCR identification of nasal and throat swabs following immunization with low-, middle-, and high-dose groups.** (**A-F**). Nasal and throat swabs were collected from each pig in the low-, middle-, and high-dose groups on day 3 (**A-B**), day 7 (**C-D**) and day 10 (**E-F**). PCR using primers P*nap*-F/*apxIIIA*-R.

**Additional file 4. PCR identification of lung and tonsil tissues collected at necropsy from low and high dose groups.** (**A-B**). Bacterial isolation and PCR identification were performed on lung tissues from different lobes and tonsil tissues from each pig in the low-, high-dose groups, using the primers P*nap*-F/*apxIIIA*-R.

## Data Availability

No datasets were generated or analyzed during the current study.
