# Supplementary material for: WH01-3A: a DIVA-compliant, ApxIA, ApxIIA, and ApxIIIA expressing Actinobacillus pleuropneumoniae live attenuated vaccine strain that protects mice and pigs against homologous and heterologous serovars
Source: Vet Res. 2026 Jul 30;57:141. doi: 10.1186/s13567-026-01819-6 (PMC13422351; doi:10.1186/s13567-026-01819-6)
Supplement: Supplementary file 1 — Additional file 1. Bacterial strains, plasmids and primers used in this study. [file 13567_2026_1819_MOESM1_ESM.docx]

**Additional file 1. Bacterial strains, plasmids and primers used in this study.**

| **Strain/plasmid/primer** | **Characteristics and/or sequences** | **Source** |
| --- | --- | --- |
| **Strains** |  |  |
| *A. pleuropneumoniae* WH01 | Clinical strain of *A. pleuropneumoniae* serovar 1 isolated from a pig farm in Hubei Province, China | This study |
| *A. pleuropneumoniae* WH01 Δ*apxIV* | *A. pleuropneumoniae* WH01 *apxIV* deleted mutant | This study |
| *A. pleuropneumoniae* WH01 Δ*apxIV*Δ*apxIC* | *A. pleuropneumoniae* WH01 *apxIV*/*apxIC* deleted mutant | This study |
| *A. pleuropneumoniae* WH01 Δ*apxIV*Δ*apxIC*Δ*apxIIC* | *A. pleuropneumoniae* WH01 *apxIV*/*apxIC*/*apxIIC* deleted mutant | This study |
| *A. pleuropneumoniae* WH01-3A | *A. pleuropneumoniae* WH01 *apxIV*/*apxIC*/*apxIIC* deleted and P*nap*/*apxIIIA* inserted mutant | This study |
| *A. pleuropneumoniae* 202203179 | Clinical strain of *A. pleuropneumoniae* serovar 5 isolated from a pig farm in Jiangxi Province, China | This study |
| *A. pleuropneumoniae* XB2T-56 | Clinical strain of *A. pleuropneumoniae* serovar 15 isolated from a pig farm in Hubei Province, China | This study |
| *A. pleuropneumoniae* JL03 | Reference strain of *A. pleuropneumoniae* serovar 3 | [31] |
| *A. pleuropneumoniae* AP76 | Reference strain of *A. pleuropneumoniae* serovar 7 | Dr P. Blackall |
| *A. pleuropneumoniae* D13039 | Reference strain of *A. pleuropneumoniae* serovar 10 | Dr P. Blackall |
| *E. coli* β2155 | Transconjugation donor. *thrB1004 pro thi strA hsdS lacZ*Δ*M15(F’ lacZ*Δ*M15lacI^q^ traD36 proA*^+^ *proB*^+^) *dap*::*erm recA*::*RP4-2-tet*::*Mu-km λpir*, Erm^r^ Tet^r^ Kan^r^ | [33] |
| *E. coli* BL21(DE3) | Expression host. F^-^ *ompT* *hsdS*(r_B_^-^ m_B_^-^) *gal dcm* (DE3) | Vazyme Biotech |
| **Plasmid** |  |  |
| pEMOC2 | Transconjugation vector: ColE1 *orimob*RP4 *sacB*, Amp^r^ Chl^r^ | [32] |
| pEMOC2*-*Δ*apxIV* | pEMOC2 carrying the upstream and downstream regions of *apxIV* operon | This study |
| pEMOC2*-*Δ*apxIC* | pEMOC2 carrying the upstream and downstream regions of *apxIC* operon | This study |
| pEMOC2*-*Δ*apxIIC* | pEMOC2 carrying the upstream and downstream regions of *apxIIC* operon | This study |
| pEMOC2*-*P*nap*/*apxIIIA* | pEMOC2 carrying the upstream of Δ*apxIV* operon, P*nap*, *apxIIIA* and downstream of Δ*apxIV* operon | This study |
| pQE80L-ApxIA | pQE80L carrying the *apxIA* gene | Our laboratory |
| pET-32a-ApxIIA | pET-32a carrying the *apxIIA* gene | Our laboratory |
| pQE80L-ApxIIIA | pQE80L carrying the *apxIIIA* gene | Our laboratory |
| **Primers (5’-3’)** |  |  |
| *apxIV* -1 | GGCCCCCCCCTCGAGGTCGACGCGATATCCAACTTACTCTTTCGC | This study |
| *apxIV* -2 | TTCGTATGTAGGATTGCCGCAATTATCAAA | This study |
| *apxIV* -3 | GCGGCAATCCTACATACGAAAGAAATCGGCACAG | This study |
| *apxIV* -4 | GATCCCACCGCGGTGGCGGCCGCTACTGCGACAGAATTATTGGAAGC | This study |
| *apxIV-*UF | TGTTGGTCTTAGGTGTCGTT | This study |
| *apxIV-*DR | GTTTCGGTTTACGCTTCTGG | This study |
| Δ*apxIV-* F | TGGCACTGACGGTGATGA | This study |
| Δ*apxIV-* R | GGCCATCGACTCAACCAT | This study |
| *apxIC* -1 | GGCCCCCCCCTCGAGGTCGACAGCGCGAAATCAACGAATAATT | This study |
| *apxIC* -2 | AGCCATGTTGTCTCCTCATCCCCCCAGATTTATAACAA | This study |
| *apxIC* -3 | GATGAGGAGACAACATGGCTAACTCTCAG | This study |
| *apxIC* -4 | GATCCCACCGCGGTGGCGGCCGCCCAACACCTGCGGAAGCC | This study |
| *apxIC-*UF | AATCAACAAAATGCCGCGAA | This study |
| *apxIC-*DR | AATTCTTTCGCTAGTGCGGT | This study |
| Δ*apxIC-* F | AGAGGTGGCATGGTTATGG | This study |
| Δ*apxIC-* R | AGCTCACTCATTAATTCAAAGTGAT | This study |
| *apxIIC* -1 | GGCCCCCCCCTCGAGGTCGACGCCGCTATTAGGCGATTTAGTC | This study |
| *apxIIC* -2 | AAAGATACCCTCCCATTCCCTAAAAAATG | This study |
| *apxIIC* -3 | GGGAATGGGAGGGTATCTTTAAATGATCAATTATATAAAGGAGACT | This study |
| *apxIIC* -4 | GATCCCACCGCGGTGGCGGCCGCGTTCCTGTCTCACGGTGAAAATC | This study |
| *apxIIC-*UF | TTTCCAAGTAAGTTATGCCAGTT | This study |
| *apxIIC-*DR | TCCATTTCCTTGACCCGAA | This study |
| Δ*apxIIC-* F | TGCTTGGTTATGGGCAAATTC | This study |
| Δ*apxIIC-* R | AGCTGTTATTAACTCTTGCTCATAC | This study |
| Δ*apxIV-1* | ggcccccccctcgaggtcgacATGTTCCAAAAAGTGTCGGTACC | This study |
| Δ*apxIV-2* | accgaacatcTAGGATTGCCGCAATTATCAAA | This study |
| Δ*apxIV-3* | CATACGAAAGAAATCGGCACAG | This study |
| Δ*apxIV-4* | gatcccaccgcggtggcggccgcTCCGCATTAACGTACACAACGG | This study |
| *Pnap* -1 | GGCAATCCTAGATGTTCGGTTTGGATTTTTGC | This study |
| *Pnap* -2 | ACTCATAACACACCTACGGCAAAGAAAAC | This study |
| *apxIIIA*-1 | GCCGTAGGTGTGTTATGAGTACTTGGTCAAGCATGTTAGC | This study |
| *apxIIIA*-2 | GTGCCGATTTCTTTCGTATGTTAAGCTGCTCTAGCTAGGTTAC | This study |
| *apxIIIA*-F | CGAAGCTGGTACTATTGGTG | This study |
| *apxIIIA*-R | CGTTGACGTTTGGCTATTTG | This study |
| P*nap-*F | TTGGAGTGATGAAAGCGGAT | This study |
| *apxIIIA-*R | TTGTTCAGTCGCTTTGGTTC | This study |
| pEMOC2-testF | GTTATTGGTGCCCTTAAACGCC | This study |
| pEMOC2-testR | GCCAAGCCCGCCGATG | This study |
| *apxIV*-F | TGGCACTGACGGTGATGA | This study |
| *apxIV*-R | GGCCATCGACTCAACCAT | This study |
| AP1F | CTGGAGTAATTACGGCGACTATTCC | This study |
| AP1R | AGGAGAAGCTAGTAGTACTTGCATTTTC | This study |
| AP15F | GCAACTTGGAGAACATGGTTAAATCAAG | This study |
| AP15R | CAACCCTCCAATGTAAGCGAAGG | This study |

Chl^r^, chloramphenicol resistant; Kan^r^, kanamycin resistant; Erm^r^, erythromycin resistant; Tet^r^, tetracycline; Amp^r^, ampicillin resistant.
