# Supplementary figures and images for: WH01-3A: a DIVA-compliant, ApxIA, ApxIIA, and ApxIIIA expressing Actinobacillus pleuropneumoniae live attenuated vaccine strain that protects mice and pigs against homologous and heterologous serovars
Source: Vet Res. 2026 Jul 30;57:141. doi: 10.1186/s13567-026-01819-6 (PMC13422351; doi:10.1186/s13567-026-01819-6)

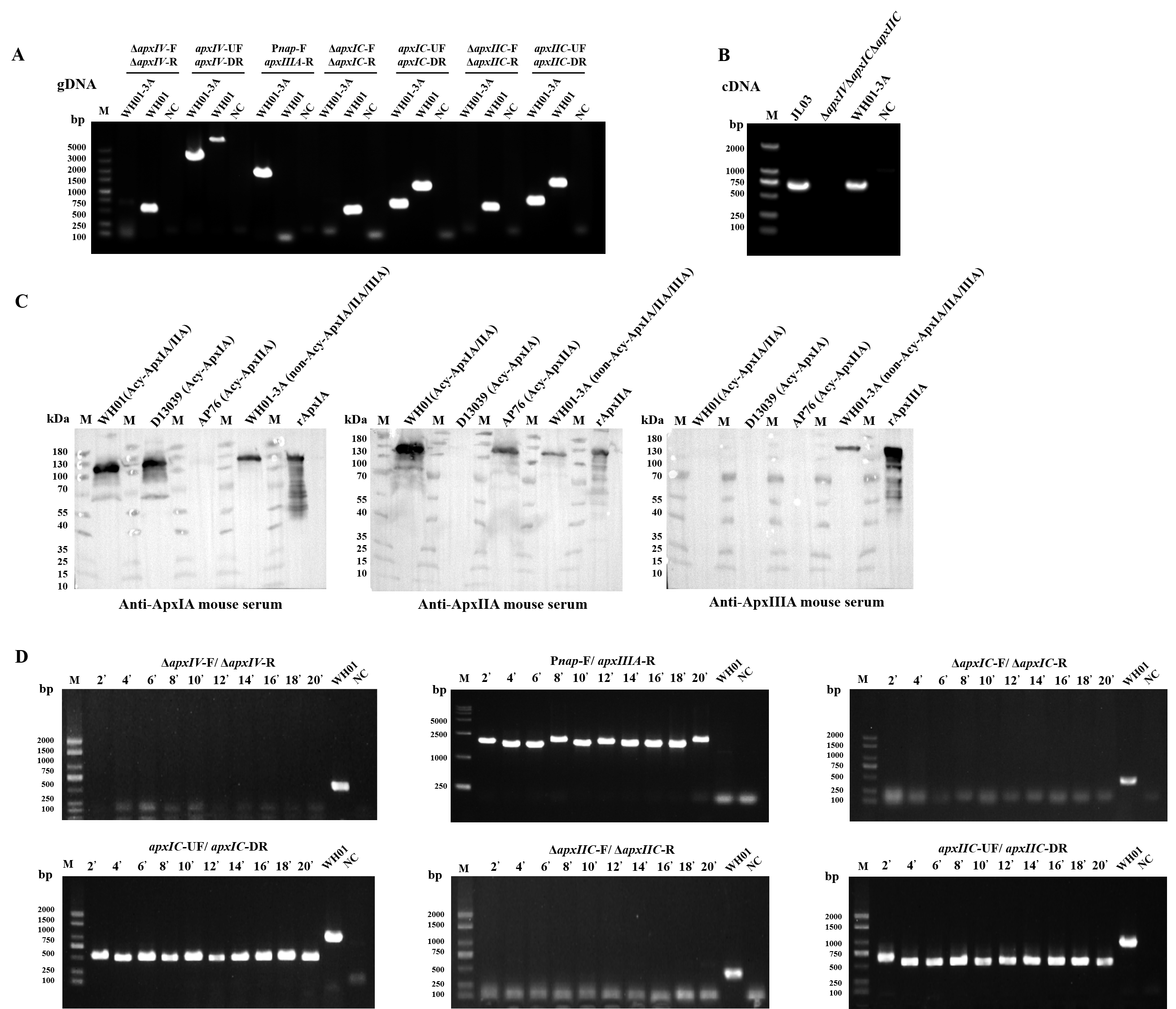

Supplement: Supplementary file 2 — Additional file 2. Identification of the WH01-3A strain. (A). Genomic DNA was extracted from WH01-3A and WH01. Primers ΔapxIV-F/R amplified an internal fragment of the apxIV gene. Primers apxIV-UF/DR amplified the upstream and downstream regions of apxIV. Primers Pnap-F/apxIIIA-R amplified an internal fragment of the inserted gene. Primers ΔapxIC-F/R amplified an internal fragment of the apxIC operon. Primers apxIIC-UF/DR amplified upstream and downstream regions of apxIIC. (B). RNA was extracted from A. pleuropneumoniae JL03, ΔapxIVΔapxICΔapxIIC, and WH01-3A , and cDNA was synthesized by RT-PCR after gDNA removal. PCR identification with the primer apxIIIA-F/apxIIIA-R. (C). The expression of native toxins non-acylated ApxIA, ApxIIA, and ApxIIIA in the WH01-3A supernatant was analyzed by western blotting. Anti-ApxIA, ApxIIA, or ApxIIIA mouse serum were used separately as primary antibodies, with the corresponding recombinant proteins rApxIA, rApxIIA, or rApxIIIA serving as positive controls. To verify the specificity of the detection method, A. pleuropneumoniae strains with distinct toxin secretion profiles were tested in parallel: serovar 1 strain WH01 secretes acylated ApxIA, ApxIIA proteins, serovar 7 strain AP76 secretes only acylated ApxIIA protein, and serovar 10 strain D13039 secretes only acylated ApxIA protein. Following SDS-PAGE and transfer to a PVDF membrane, proteins were probed with Anti-ApxIA, ApxIIA, or ApxIIIA mouse serum (1:5000, 4°C, overnight) and subsequently with HRP-conjugated goat anti-mouse IgG (1:10,000, 37°C, 1 h). Detection was performed by enhanced chemiluminescence. (D). Genetic stability of the knockout or inserted genes during serial passage. Genomic DNA was extracted from even-numbered (2-20) passages of WH01-3A, with WH01 genomic DNA used as a control. [file 13567_2026_1819_MOESM2_ESM.tif]

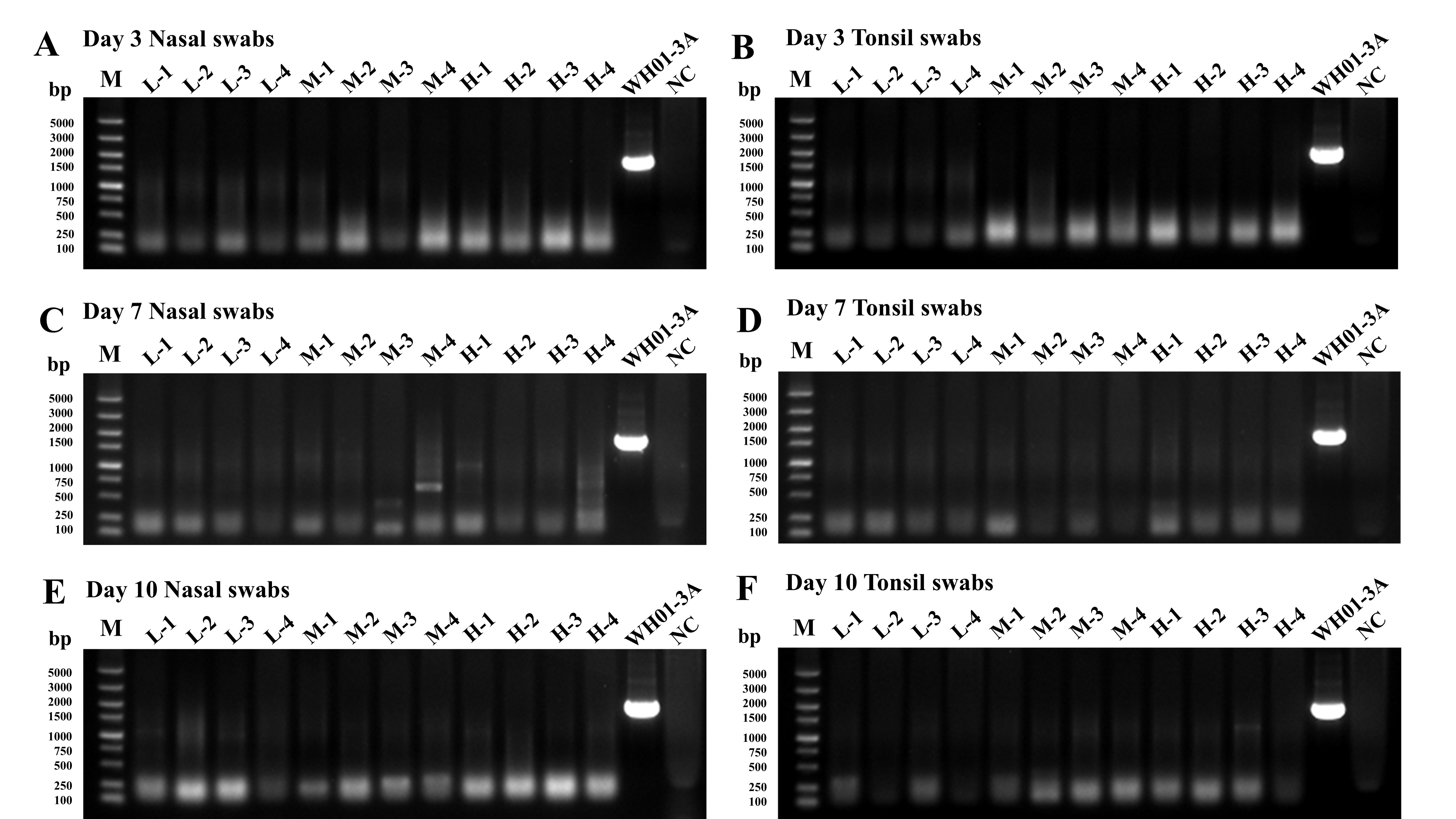

Supplement: Supplementary file 3 — Additional file 3. PCR identification of nasal and throat swabs following immunization with low-, middle-, and high-dose groups. (A-F). Nasal and throat swabs were collected from each pig in the low-, middle-, and high-dose groups on day 3 (A-B), day 7 (C-D) and day 10 (E-F). PCR using primers Pnap-F/apxIIIA-R. [file 13567_2026_1819_MOESM3_ESM.tif]

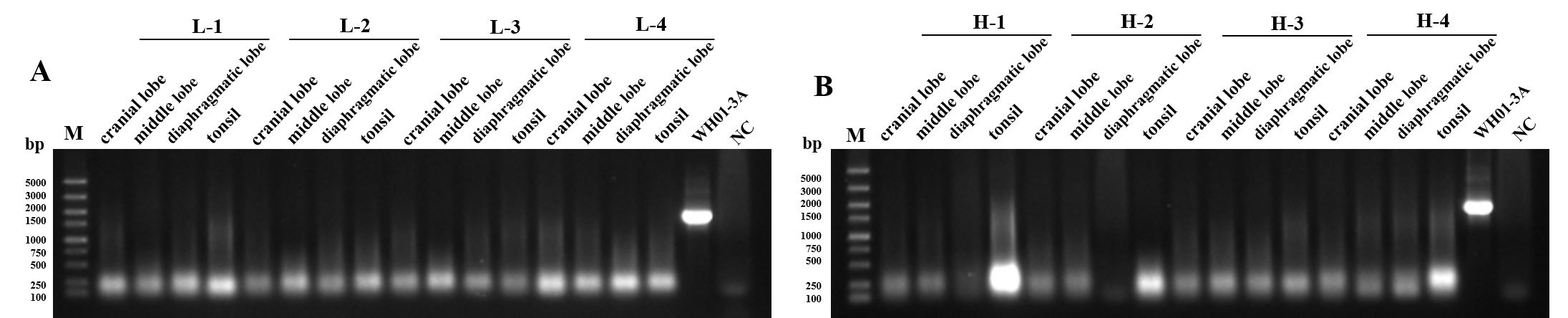

Supplement: Supplementary file 4 — Additional file 4. PCR identification of lung and tonsil tissues collected at necropsy from low and high dose groups. (A-B). Bacterial isolation and PCR identification were performed on lung tissues from different lobes and tonsil tissues from each pig in the low-, high-dose groups, using the primers Pnap-F/apxIIIA-R. [file 13567_2026_1819_MOESM4_ESM.tif]
